# Mitochondrial protein alterations in vascular dementia: evidence from Mendelian randomization, transcriptomics, and a chronic hypoperfusion model

**DOI:** 10.3389/fneur.2026.1794851

**Published:** 2026-07-09

**Authors:** Qian Liu, Huizhong Tan, Keke Tong, Ruhai Luo, Hanquan Li, Feng Qiu, Shiliang Wang, Le Xie, Xiuli Zhang, Dahua Wu

**Affiliations:** 1 Graduate School of Hunan University of Chinese Medicine, Changsha, Hunan, China; 2 Department of Neurology, Hunan Hospital of Integrated Traditional Chinese and Western Medicine, Changsha, Hunan, China; 3 Institute of Innovation and Applied Research, Hunan University of Chinese Medicine, Changsha, Hunan, China

**Keywords:** bioinformatics, Gene Expression Omnibus, analysis, Mendelian randomization, mitochondrial dysfunction, vascular dementia

## Abstract

**Objective:**

Mitochondrial dysfunction is a key pathological feature of vascular dementia (VaD), yet the specific systemic and localized proteins involved remain unclear. We aim to utilize Mendelian randomization (MR) of plasma proteomics and Gene Expression Omnibus (GEO) datasets to identify systemic circulating biomarkers associated with VaD risk, and subsequently use *in vivo* models to investigate whether these systemic signals correspond to actual neuropathological mitochondrial changes in the brain.

**Methods:**

We first identified candidate proteins associated with VaD risk through a two-sample MR analysis of plasma proteomics and GWAS data. Candidate genes were then assessed for differential expression using the GEO dataset GSE122063. Finally, the proteins were validated in a Bilateral Common Carotid Artery Occlusion (2-VO) rat model by evaluating pathological features and measuring its expression levels via Western Blot.

**Results:**

Mendelian randomization analysis identified four proteins nominally linked to VaD: protective factors (AIFM1, COX5B) and risk factors (NDUFV2, NUDT5). However, cross-referencing these genetic predictions with GEO transcriptomics (GSE122063) and a 2-VO rat model revealed distinct tiers of evidentiary support. COX5B emerged as the most robust targets, demonstrating unidirectional consistency across all three analytical layers. NUDT5 showed partial consistency, supported by genetic and animal data, though its transcriptomic alteration fell short of the threshold. Conversely, AIFM1 and NDUFV2 displayed clear directional contradictions between the genetic/transcriptomic data and actual *in vivo* protein expression. Notably, while donepezil improved VaD pathology, it did not alter the expression of these proteins.

**Conclusion:**

Rather than universally validating all four genetic candidates, this rigorous multi-layer triangulation specifically pinpoints the dysregulation of COX5B as high-confidence, consistent drivers of mitochondrial impairment in VaD. Acknowledging the inconsistent complexities of AIFM1, NDUFV2, and NUDT5. Therapeutic strategies targeting the cleanly triangulated protein offer a more reliable, disease-modifying approach for VaD intervention.

## 1 Introduction

Vascular dementia (VaD), recognized as the second most prevalent form of dementia following Alzheimer’s disease, poses an escalating public health concern in the context of a globally aging population (1). This condition arises from chronic cerebral hypoperfusion attributed to cerebrovascular disease, leading to a progressive decline in cognitive function and a marked reduction in quality of life, which in turn results in significant socioeconomic and caregiving challenges (2). Despite its widespread occurrence, therapeutic options for VaD remain notably limited. Current treatments, such as the acetylcholinesterase inhibitor donepezil, primarily provide symptomatic relief without addressing the underlying neurodegenerative processes (3, 4). Consequently, understanding the molecular mechanisms underlying VaD pathogenesis is of paramount importance for the development of novel, disease-modifying therapeutic targets.

Chronic cerebral ischemia drives VaD pathology by triggering energy failure, oxidative stress, and neuronal death (5, 6). While mitochondrial dysfunction, including metabolic impairment, ROS accumulation, and defective mitophagy, is a well-established hallmark of the disease (7–9), the specific mitochondrial proteins initiating this cascade remain unidentified. Crucially, evidence establishing a direct relationship between specific mitochondrial proteins and VaD is still missing.

Establishing this causal relationship presents methodological challenges. While traditional observational epidemiological studies can identify associations, they are often confounded by various factors, such as lifestyle or drug interactions, which complicates the ability to draw conclusions. Mendelian Randomization (MR) analysis offers a novel research approach. This method employs randomly assigned genetic variants, particularly quantitative trait loci (pQTLs) that influence protein levels, as instrumental variables. It simulates randomized controlled trials at the genetic level, effectively overcoming the limitations of traditional methodologies (10). This approach provides genetic evidence for inferring relationships between exposure (protein) and outcome (VaD). Building on the genetic predictions derived from MR, subsequent cross-referencing across multiple biological strata is essential. This involves corroborating findings at the transcriptomic level using public databases (e.g., GEO) and providing empirical biological context through *in vivo* animal models. Such a multi-layered approach aids in constructing a tiered evidentiary framework for identifying effective therapeutic targets (11).

**Figure 1:**
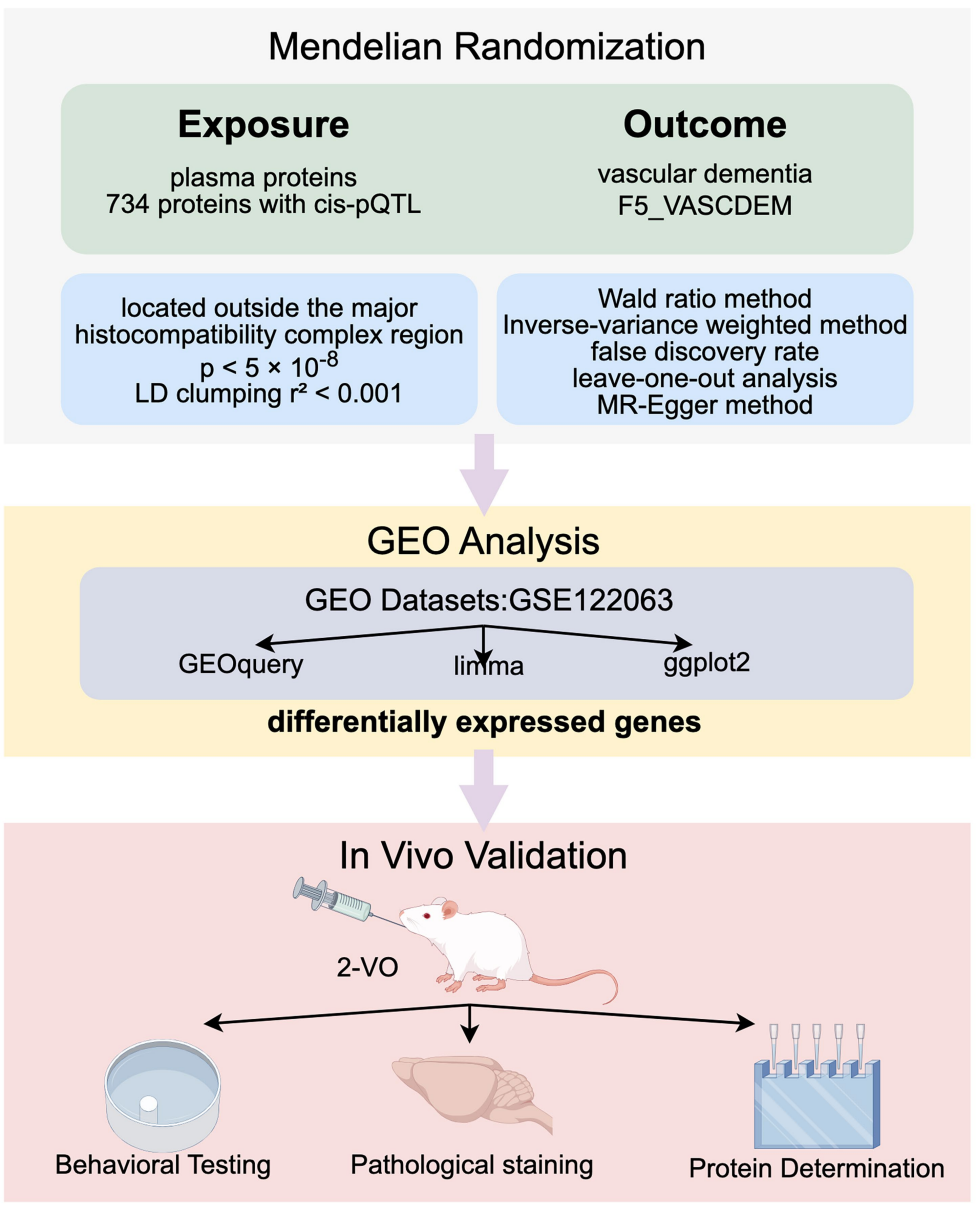
Flowchart for identifying pathogenic mitochondrial proteins in vascular dementia. Created by the authors using Figdraw (www.figdraw.com; Copyright Code: ROWSW28888).

While previous Mendelian randomization (MR) studies in dementia have predominantly investigated broad clinical risk factors or utilized generalized omics approaches, the specific causal role of mitochondrial protein dysregulation in VaD pathogenesis has remained largely unexplored. To address this gap, our study employed genetic screening, transcriptomic exploration, and *in vivo* validation to identify and evaluate mitochondrial proteins associated with VaD. We first screened candidate proteins using large-scale plasma proteomics and genome-wide association study (GWAS) data for VaD via two-sample MR analysis. Subsequently, we examined the transcriptional level changes of these candidate genes in brain tissue from VaD patients using the Gene Expression Omnibus (GEO) database. Finally, we assessed the expression of these candidate proteins *in vivo* by establishing a classical bilateral permanent ligation of the Bilateral Common Carotid Artery Occlusion (2-VO) rat model (Figure 1). By shifting the focus specifically to mitochondrial targets, our study provides reliable biomarkers for the early diagnosis and prognostic evaluation of VaD, and offers scientifically grounded potential targets for the development of novel and precise therapeutic agents.

## 2 Materials and methods

### 2.1 Data sources of MR

For this two-sample Mendelian randomization study, we sourced summary-level data for exposure (plasma proteins) and outcome (vascular dementia) from independent, non-overlapping GWAS. The exposure data for plasma proteins were obtained from a comprehensive meta-analysis published in *Nature Genetics* (12), which integrated five large-scale GWAS datasets (13–17). To define the mitochondrial protein subset, we relied on the functional and cellular component annotations provided by the IEU Open GWAS project database. By cross-referencing the initial plasma proteins with these annotations, we identified 66 proteins explicitly classified as mitochondrial-related, which subsequently served as our specific candidate pool. We selected single nucleotide polymorphisms (SNPs) as instrumental variables (IVs) based on the following stringent criteria: (i) located outside the major histocompatibility complex (MHC) region; (ii) reaching genome-wide significance (*p* < 5 × 10^−8^); (iii) functioning as a cis-acting protein quantitative trait locus (cis-pQTL); and (iv) being independent (LD clumping r^2^ < 0.001). To mitigate bias from weak instruments, the F-statistic was calculated for each SNP, and only those with *F* > 10 were retained (18). Palindromic SNPs were systematically removed. The outcome data, GWAS summary statistics for VaD, were sourced from the FinnGen consortium (ID: F5_VASCDEM). This dataset includes 3,624 cases and 475,484 controls of European ancestry. By incorporating these specific case and control counts, we ensure transparency regarding the statistical framework of our MR analysis. This cohort consists of individuals of European ancestry and is publicly available. Crucially, there was no sample overlap between the exposure and outcome cohorts.

### 2.2 MR analysis and sensitivity analyses

The analysis adheres to the three core assumptions of MR. The “TwoSampleMR” package in R was utilized. SNPs in the dataset that are mismatched or contain missing values will be automatically removed. For proteins with a single cis-pQTL, the Wald ratio method was used. For proteins with multiple IVs, the random-effects inverse-variance weighted (IVW) method was the primary analysis. FDR-adjusted *p*-values were additionally calculated for comprehensive transparency. A series of sensitivity analyses were performed to assess the robustness of our results. For exposures with multiple IVs, we conducted a leave-one-out analysis by iteratively removing each SNP to ensure the causal estimate was not driven by a single variant. Heterogeneity among instrumental variables was assessed using Cochran’s Q statistic derived from the IVW method. Furthermore, potential directional horizontal pleiotropy was evaluated using the MR-Egger intercept test.

### 2.3 Bioinformatics exploration

To explore the expression of the four candidate mitochondrial protein genes identified by MR analysis, we downloaded and analyzed the microarray dataset GSE122063 from the GEO (19). This dataset, generated using the GPL16699 platform, comprises transcriptomic data from human post-mortem brain tissues, specifically targeting the frontal and temporal cortices. The cohort includes a total of 17 samples, consisting of 11 healthy controls and 6 patients diagnosed with VaD. We retrieved raw data using the R package GEOquery and performed background correction and normalization with the limma package to identify differentially expressed genes (DEGs) between VaD and control samples. Results were visualized as volcano plots using the ggplot2 package.

### 2.4 Animals

Male Sprague–Dawley (SD) rats (n = 38, weighing 200–220 g) were purchased from Hunan Slike Jingda Laboratory Animal Co., Ltd. Animal modeling and experimental procedures in this study adhered to the National Institutes of Health Guidelines for the Care and Use of Laboratory Animals. SD rats were housed at the Animal Experiment Center of Hunan University of Chinese Medicine (Certificate of Quality No.: ZS-202504010023). This study received ethical approval from the Animal Ethics Committee of Hunan University of Chinese Medicine (Ethics Review No.: SLBH-202503050002).

### 2.5 Randomization, allocation concealment, and blinding

This study utilizes a random number table for all allocations. Considering the risk of death from gavage and modeling, 11 rats were randomly assigned to the sham group, and 27 underwent 2-VO surgery. Rats failing to exhibit significant cognitive impairment, defined *a priori* as an average escape latency that did not exceed the mean plus 1.5 standard deviations (mean + 1.5 SD) of the Sham group, were classified as modeling failures and excluded (*n* = 5). The remaining 22 VaD rats were randomized at a 1:1 ratio into the model and donepezil groups. Over the 28-day gavage phase, one rat per group died from gavage-related complications or the delayed effects of surgery. Therefore, a final cohort of 30 rats (*n* = 10 per group) was retained for subsequent evaluations.

To minimize potential selection and performance biases, strict allocation concealment and multi-stage blinding protocols were enforced. All random allocation sequences (encompassing the initial surgical assignments, the MWM-based screening, and the secondary randomization) were generated independently by Le Xie. The interventions (saline and donepezil) were prepared in identical volumes and packaging, masked with numerical codes by Le Xie prior to administration. Although the surgeons (Ruhai Luo and Hanquan Li) were aware of the specific procedures during modeling, they were strictly excluded from all post-operative interventions and outcome evaluations. Post-operative treatments were managed by Keke Tong and Shiliang Wang, who were completely blinded to the group allocations. Subsequent behavioral testing and biochemical assays were conducted by Huizhong Tan, Qian Liu, and Feng Qiu, with no knowledge of the group allocations. Finally, the ultimate data evaluation and statistical analyses were executed by Xiuli Zhang and Dahua Wu, with all group codes kept strictly concealed until the conclusion of the study.

### 2.6 VaD model establishment and drug administration

By employing the internationally recognized bilateral permanent occlusion of the common carotid arteries, the 2-VO model was established. The left common carotid artery of the rats was exposed and ligated after they were anesthetized with 2% sodium pentobarbital. Adequate anesthetic depth was confirmed by the absence of pedal withdrawal and corneal reflexes before surgery. Specifically, 2% sodium pentobarbital was administered by intraperitoneal injection at a dose of 2 mL/kg. Seven days later, the same procedure was performed on the right common carotid artery. The success of the 2-VO model establishment was verified using the Morris water maze (MWM) test (20). Rats assigned to the donepezil group were administered donepezil (0.45 mg/kg/day, gavage) while rats in the sham and model groups received an equivalent volume of saline. The sham surgery group underwent bilateral common carotid artery dissection without ligation. Following the water maze test, rats in the treatment group received oral gavage administration for 28 consecutive days, while the model and sham surgery groups received equal volumes of distilled water.

### 2.7 Morris water maze test

Spatial learning and memory were assessed using the MWM test. The apparatus consisted of a circular pool filled with opaque water maintained at 22 ± 1 °C. The pool was divided into four quadrants, and a hidden escape platform (diameter: 10 cm) was submerged 1 cm below the water surface and placed in the second quadrant. Rats underwent training trials for 4 consecutive days, with 4 trials per day and a 15-min interval between trials (21). In each trial, the animal was gently placed into the water facing the wall at one of four randomly assigned starting points. Rats were allowed to swim freely for 60 s to locate the hidden platform. If the rat failed to find the platform within this time, it was gently guided to the platform and allowed to remain there for 20 s to facilitate spatial learning. Escape latency, swim speed, and swim path were recorded using a video tracking system (SuperMaze, Xinruan Information Technology Co., Ltd. Shanghai). A probe trial was performed 24 h after the last training day, during which the platform was removed and the rat was allowed to swim for 60 s. The percentage of time spent in the target quadrant and the number of platform crossings were used as indices of spatial memory retention.

### 2.8 Tissue collection

Following the 28-day intervention, rats in each group were sacrificed to obtain brain tissues for downstream analyses. For histological evaluations, the designated subset of rats (*n* = 4 per group) was deeply anesthetized with sodium pentobarbital at 150 mg/kg by intraperitoneal injection and then subjected to transcardial perfusion with 4% paraformaldehyde (PFA) to obtain fixed brain tissues (6). For biochemical and Western blot analyses, the remaining subset of rats (*n* = 6 per group) was anesthetized prior to decapitation with sodium pentobarbital at 150 mg/kg by intraperitoneal injection and then euthanized by rapid decapitation without perfusion. For biochemical and Western blot analyses, the remaining subset of rats (*n* = 6 per group) was deeply anesthetized with sodium pentobarbital at 150 mg/kg by intraperitoneal injection and then euthanized by rapid decapitation without perfusion; their hippocampal tissues were immediately harvested on ice and flash-frozen in liquid nitrogen, and stored at −80 °C until use. Adequate anesthetic depth was confirmed by the absence of pedal withdrawal and corneal reflexes before perfusion or decapitation. Death was confirmed by the absence of heartbeat, spontaneous respiration, and reflex responses before tissue harvesting.

### 2.9 HE and Nissl staining

On the 28th day post-intervention, rat brains were collected for pathological evaluation using HE and Nissl staining. Following anesthesia, the brains were harvested, washed with cold saline, and fixed in 4% paraformaldehyde for 24 h. The tissues were then dehydrated, embedded in paraffin, and cut into sections 5 μm thick. For the purpose of HE staining, sections were first deparaffinized and rehydrated, then stained with hematoxylin for 5 min. A differentiation step was followed by a 1-min eosin counterstain to assess hippocampal morphology. For Nissl staining, sections underwent a 10-min staining with a 0.1% Nissl solution, followed by differentiation, dehydration, and mounting.

### 2.10 Measurement of malondialdehyde (MDA) in rat hippocampal tissue

The level of MDA, a marker of lipid peroxidation, in rat hippocampal tissue was determined using a commercial assay kit (Cat. No. A003-1, Nanjing Jiancheng Bioengineering Institute, China). Briefly, hippocampal tissues were quickly excised, weighed, and homogenized in ice-cold physiological saline to prepare a 10% (w/v) tissue homogenate. The homogenates were centrifuged at 2500–3000 rpm for 10 min at 4 °C, and the supernatant was collected for analysis. MDA reacts with thiobarbituric acid (TBA) to produce a red-colored MDA-TBA adduct, which was measured spectrophotometrically at 532 nm. Based on the standard curve, the MDA concentration was calculated and expressed in nmol/mg protein. The protein content was determined using a BCA protein assay kit (Beyotime, Shanghai, China).

### 2.11 Measurement of superoxide dismutase (SOD) in rat hippocampal tissue

Superoxide dismutase activity in rat hippocampal tissue was measured using the Biosharp Total SOD Activity Assay Kit (BL902A). Following euthanasia, hippocampal tissue was immediately harvested and homogenized in ice-cold pre-chilled physiological saline at a 1:1 tissue-to-saline ratio. The homogenate was centrifuged at 4 °C and 3,000 r/min for 10 min, and the supernatant was collected. Prepare the reaction system according to the instructions. Incubate at 37 °C for 20 min, then measure the absorbance at 450 nm. Calculate SOD activity based on the inhibition rate. Determine protein concentration using the BCA method. Express SOD activity as U/mg protein.

### 2.12 Measurement of ATP levels in rat hippocampal tissue

Adenosine triphosphate (ATP) levels in rat hippocampal tissue were quantified using a chemiluminescence-based ATP assay kit (Cat. No. A095-2-1, Nanjing Jiancheng Bioengineering Institute, Nanjing, China), according to the manufacturer’s protocol. Hippocampal tissues were rapidly dissected on ice and homogenized in the lysis buffer provided with the kit. The homogenates were centrifuged at 12,000 × g for 10 min at 4 °C, and the supernatants were collected for analysis. The assay is based on the firefly luciferase – luciferin system, in which ATP reacts with luciferin catalyzed by luciferase to produce light. The luminescence intensity is directly proportional to the ATP concentration. The chemiluminescence signal was measured using a microplate luminometer, and ATP levels were calculated from a standard curve prepared with known ATP concentrations. Results were normalized to total protein content and expressed as μmol/g protein. Protein concentration was determined using a bicinchoninic acid (BCA) protein assay kit (Beyotime Biotechnology, Shanghai, China).

### 2.13 Western blot

Pre-chilled rat hippocampal tissue was collected and thoroughly homogenized on ice after the addition of pre-chilled Mitochondria Extraction Buffer A (Epizyme, PC205). The homogenate was layered on top of Mitochondria Extraction Buffer B and centrifuged at low speed (700 g for 10 min) at 4 °C to remove nuclei and unbroken cells. Following the collection of the supernatant, it was subjected to a second centrifugation at high speed (10,000 g for 10 min) at 4 °C to precipitate and isolate the crude mitochondria. The resulting pellet was resuspended in Mitochondria Wash Buffer and layered on top of a pre-prepared mixture of Mitochondria Extraction Buffer C/D, followed by ultracentrifugation (21,000 g for 10 min) at 4 °C. The supernatant was discarded, yielding the high-purity mitochondria in the pellet. Subsequently, Mitochondria Lysis Buffer containing protease inhibitors was added to the purified mitochondrial pellet, and the protein sample obtained after thorough lysis was utilized for subsequent analysis. Electrophoresis conditions were adjusted based on molecular weight, and proteins were transferred to a polyvinylidene fluoride (PVDF) membrane. The membrane was blocked with Rapid Blocking Buffer (Becton Dickinson, China) for 1 h at room temperature on a platform shaker. It was subsequently incubated in primary antibody solutions: NUDT5 (1:2000, Thermo Fisher, MA5-426101), AIFM1 (1:2000, Thermo Fisher, MA5-15880), NDUFV2 (1:2000, Proteintech Group, 15,301-1-AP), COX5B (1:3000, Proteintech Group, 11,418-2-AP), and VDAC1 (1:1500, Epizyme, R012378) overnight. The following day, secondary antibodies (anti-mouse/rabbit HRP) (1:10000, Proteintech Group, Inc., SA00001-1, SA00001-2) were added and incubated for 1 h. To validate the suitability of VDAC1 as an internal reference under experimental stress, the raw optical densities of VDAC1 bands across all groups (resolved on the same gel) were quantified to confirm expression stability prior to normalization. Protein bands were made visible with ECL reagents from Biosharp, China, and analyzed through ImageJ software.

### 2.14 Statistical analysis

All quantitative data are presented as the mean ± standard error of the mean (SEM). Statistical analyses were performed using SPSS 26.0. Differences among groups were assessed using a one-way analysis of variance (ANOVA). Statistical analysis of escape latency over the training days was conducted using repeated measures ANOVA to evaluate the effects of time and treatment. For data exhibiting uniform variances, pairwise comparisons after the fact were performed using the LSD test. In cases of unequal variances, Dunnett’s T3 test was applied, and *p* < 0.05 was considered statistically significant.

## 3 Results

### 3.1 Identification of causal proteins associated with VaD

**Figure 2:**
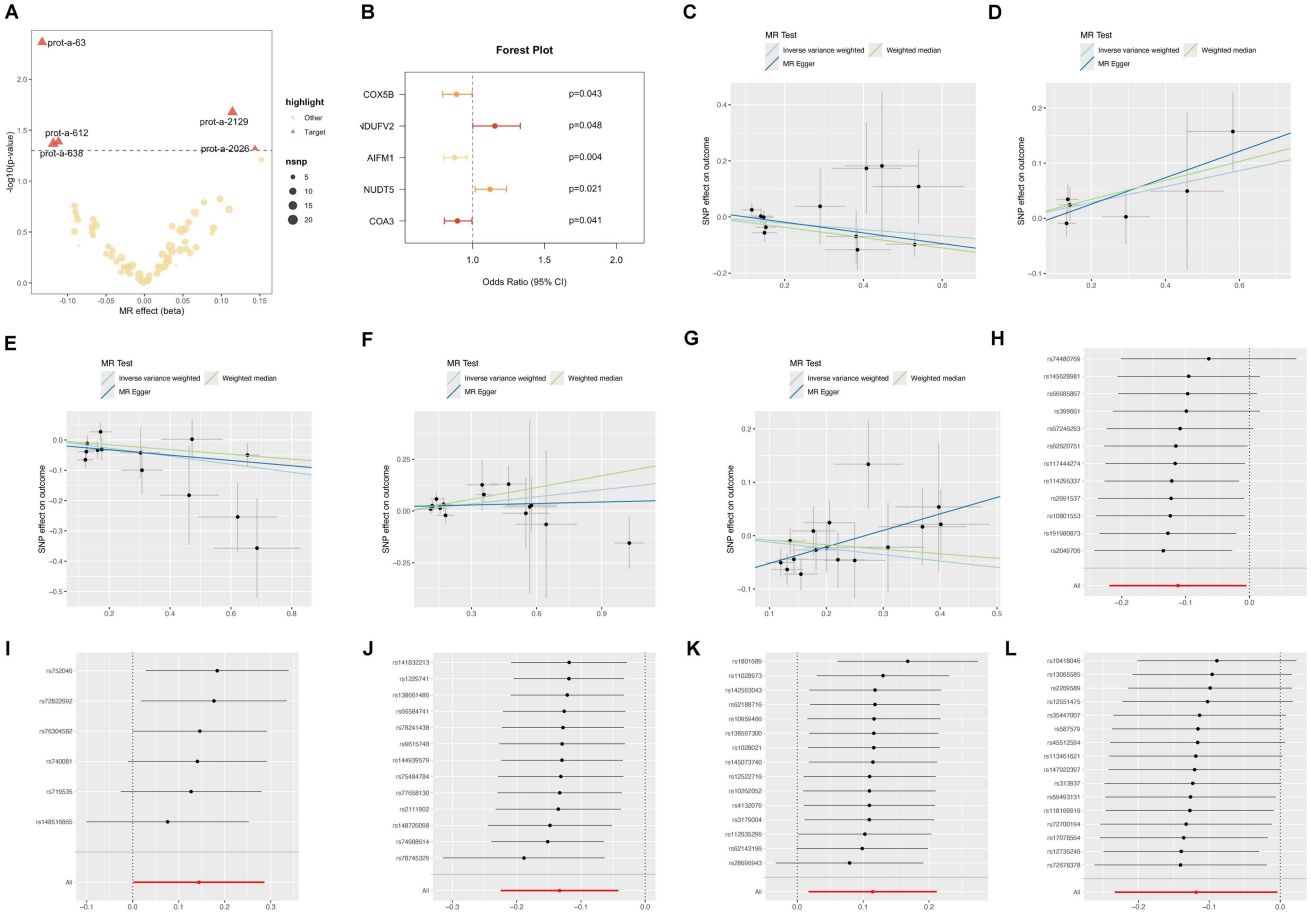
Visualization of MR study results and sensitivity analysis. **(A)** MR’s volcano chart. **(B)** Forest plot of significant mitochondrial proteins. **(C–G)** Scatter plot for COX5B, NDUFV2, AIFM1, NUDT5, and COA3. **(H–L)** Leave one out sensitivity analyses of COX5B, NDUFV2, AIFM1, NUDT5, and COA3.

We performed a Mendelian randomization study using plasma proteins as the exposure and VaD as the outcome. Our primary analysis, using the inverse variance weighted (IVW) method, identified 66 mitochondrial-related proteins, five of which showed preliminary associations with VaD: prot-a-2129 (NUDT5), prot-a-2026 (NDUFV2), prot-a-612 (COA3), prot-a-63 (AIFM1), and prot-a-638 (COX5B) (Figures 2A,B). Specifically, higher genetically predicted levels of NDUFV2 (*p* = 0.048, OR = 1.154, 95% CI: 1.001–1.331) and NUDT5 (*p* = 0.021, OR = 1.121, 95% CI: 1.017–1.1.236) were associated with increased VaD risk (Figures 2C,D), while elevated levels of COX5B (*p* = 0.043, OR = 0.888, 95% CI: 0.792–0.959), AIFM1 (*p* = 0.004, OR = 0.875, 95% CI: 0.799–0.959), and COA3 (*p* = 0.041, OR = 0.894, 95% CI: 0.803–0.995) correlated with a reduced risk of VaD (Figures 2E–G). However, after adjusting for multiple testing using the Benjamini-Hochberg FDR method across the 66 screened mitochondrial proteins, AIFM1 (*p*_FDR_ = 0.285), COA3 (*p*_FDR_ = 0.628), COX5B (*p*_FDR_ = 0.628), NDUFV2 (*p*_FDR_ = 0.628) and NUDT5 (*p*_FDR_ = 0.628) exhibited nominal significance (*p* < 0.05) but did not survive the FDR correction. For the five candidate, primary causal estimates were derived using the IVW method, supported by multiple IVs ranging from 6 to 16 SNPs. Detailed sensitivity analyses for all 66 evaluated proteins are provided in Supplementary Table 1. During this strict quality control phase, COA3 was excluded from the final candidate pool due to the presence of significant directional horizontal pleiotropy (MR-Egger intercept *p* < 0.05) and a subsequent loss of causal significance in the Weighted Median and MR-Egger model (*p* > 0.05). In contrast, the associations for the remaining four candidate proteins proved highly robust. Specifically, Cochran’s *Q* and MR-Egger intercept tests indicated no substantial heterogeneity or directional horizontal pleiotropy (all *p* > 0.05). Additionally, leave-one-out analyses confirmed that these final results were not driven by any individual SNP. Therefore, these four proteins were necessitated subsequent *in vivo* experimental validation (Figures 2H–L).

### 3.2 Transcriptomic exploration in VaD

**Figure 3:**
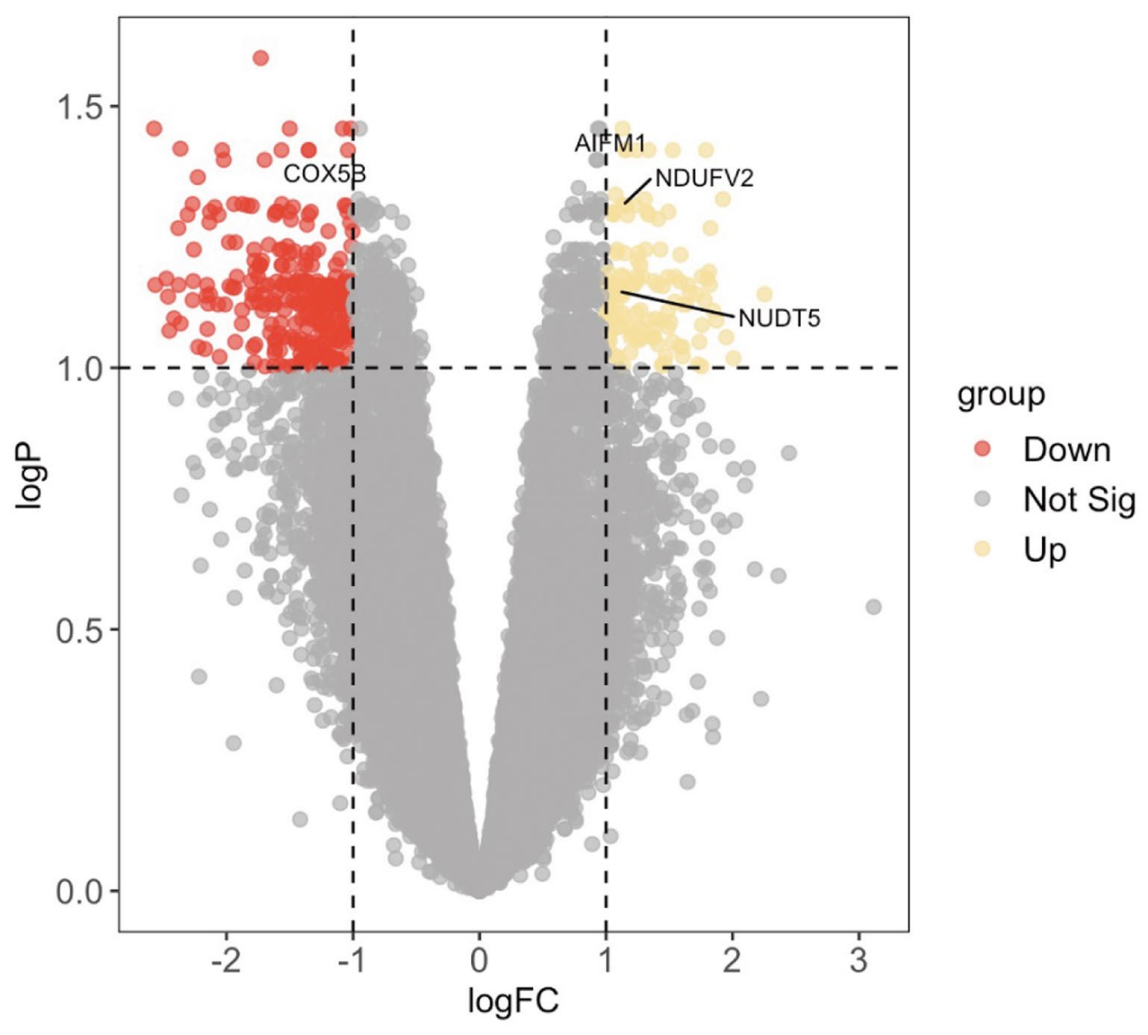
Volcano plot for GEO analysis. Positive logFC indicates up-regulation, negative logFC indicates down-regulation.

To explore the candidate genes identified through MR analysis at the transcriptomic level, we conducted an analysis of the GSE122063 dataset. The differential expression profile, strictly utilizing the standard contrast direction (VaD vs. Control, where a positive Log_2_FC denotes up-regulation in VaD), is illustrated in the volcano plot (Figure 3). Upon examining our four candidate genes, we observed that AIFM1 and NDUFV2 were significantly up-regulated (Log_2_FC > 1). NUDT5 also exhibited a positive up-regulation trend, although it did not achieve statistical significance. In contrast, COX5B was significantly down-regulated (Log_2_FC < −1).

### 3.3 Pathological characterization of the VaD rats

**Figure 4:**
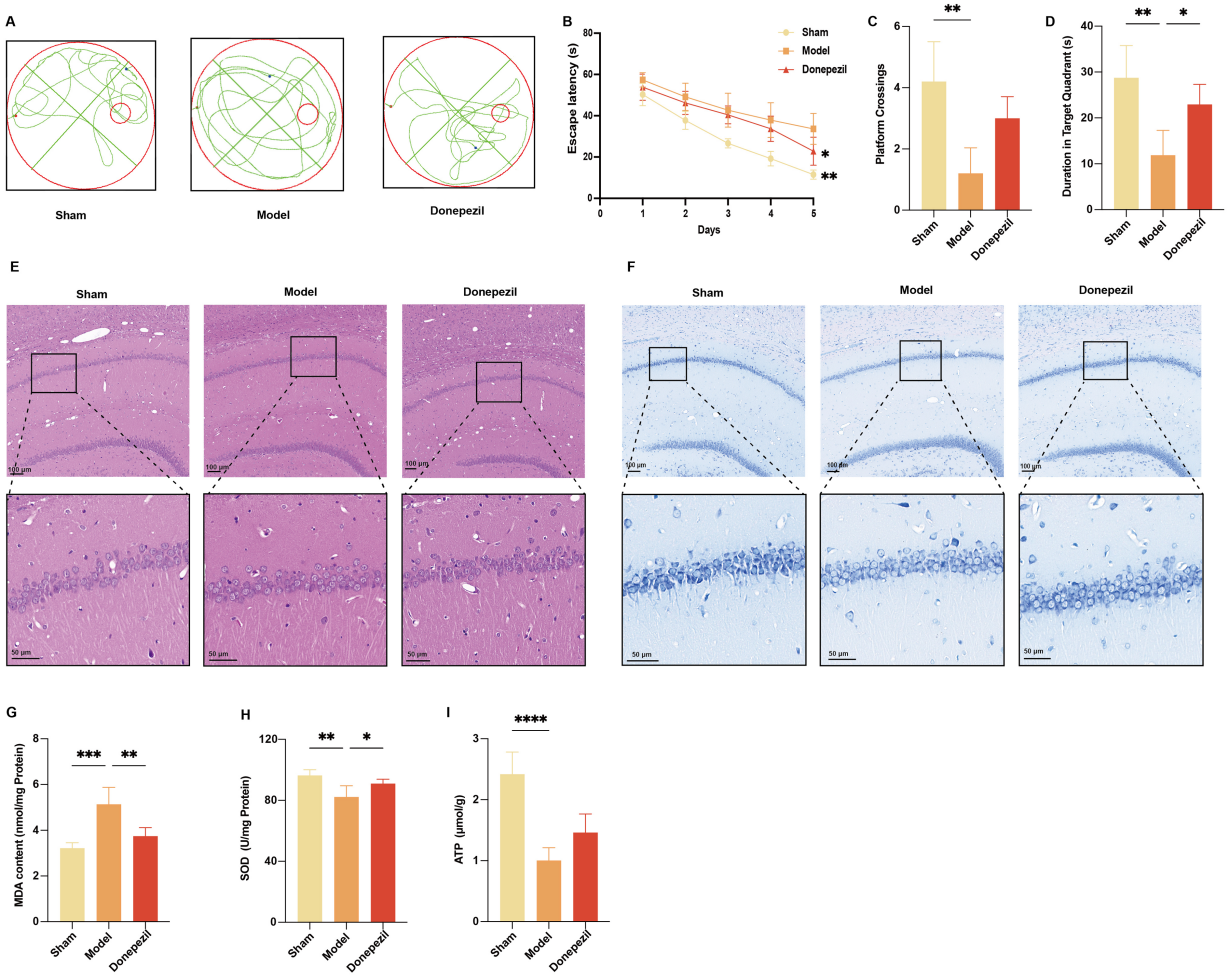
The pathological features of rats. **(A)** Escape latency trajectory in the Morris water maze test. **(B)** Escape latency in the MWM test. **(C)** Number of platform crossings in the MWM test. **(D)** Duration in target quadrants in the MWM test. **(E)** Hematoxylin and eosin staining of rat hippocampal CA1 region. **(F)** Nissl staining of rat hippocampal CA1 region. **(G)** MDA content in rat hippocampal CA1 region. **(H)** SOD content in rat hippocampal CA1 region tissue. **(I)** ATP content in rat hippocampal CA1 region. *n* = 5, *p < 0.05, **p < 0.01, ***p < 0.001, ****p < 0.0001.

To verify the potential functions of our candidate genes in vivo, we first established a rat model of VaD via bilateral common carotid artery occlusion (2-VO). We evaluated the pathological features of this model and benchmarked them against the effects of a positive control drug, donepezil (22). We first evaluated cognitive function using the Morris water maze. Compared to the sham group, the 2-VO model rats exhibited significant deficits in spatial learning and memory, as evidenced by prolonged escape latency (*p* < 0.01) and fewer platform crossings (*p* < 0.01). Treatment with donepezil effectively ameliorated these cognitive impairments, significantly reducing the escape latency (*p* < 0.05) and increasing platform crossings (*p* < 0.05) (Figures 4A–D). Consistent with the behavioral outcomes, histopathological analysis revealed severe neuronal damage in the hippocampal CA1 region of the model rats. HE staining showed that while sham-operated rats had neatly arranged neurons, the 2-VO group displayed cellular disorganization, enlarged intercellular spaces, and abundant pyknotic nuclei indicative of necrosis (Figure 4E). Similarly, Nissl staining demonstrated a dramatic loss and dissolution of Nissl bodies in the model group, with neurons appearing shrunken and vacuolated (Figure 4F). Importantly, donepezil treatment markedly attenuated this neuronal damage, restoring a more organized cellular structure and preserving Nissl body integrity. Given that oxidative stress and metabolic dysfunction are core features of VaD, we measured related indicators in the hippocampus. The model rats showed a significant decrease in the activity of the antioxidant enzyme SOD (*p* < 0.01) and a concurrent increase in the lipid peroxidation marker MDA (*p* < 0.01) (Figures 4G,H). Furthermore, ATP levels were sharply reduced (*p* < 0.01) (Figure 4I), confirming a state of severe oxidative stress and energy failure. Following donepezil treatment, this imbalance was reversed, manifested by a significant rebound in SOD levels (*p* < 0.05) and a significant reduction in MDA levels (*p* < 0.01).

### 3.4 *In vivo* validation of candidate protein expression in the VaD rat hippocampus

**Figure 5:**
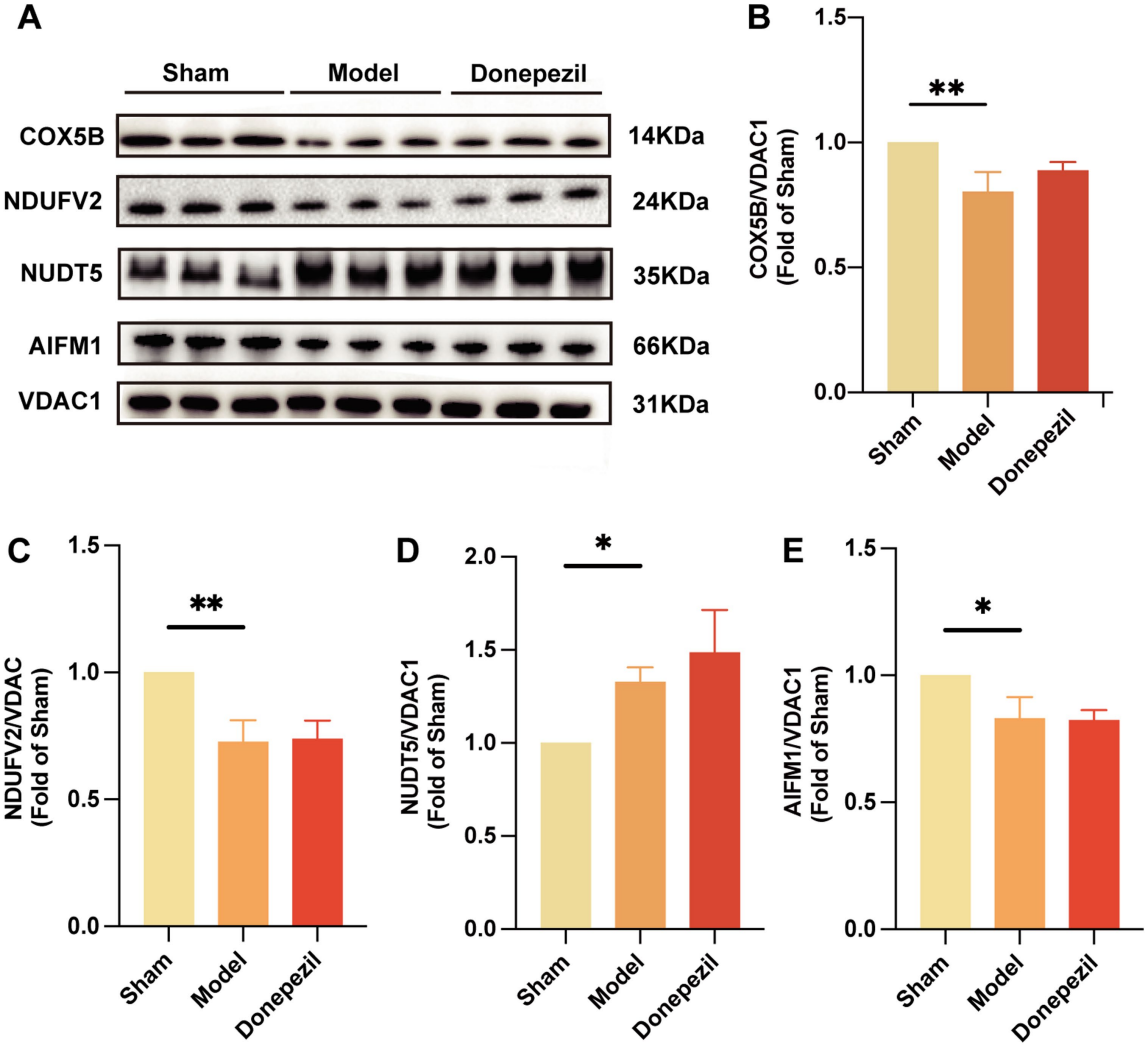
Mitochondrial protein expression. **(A)** The representative Western blotting of COX5B, NDUFV2, NUDT5, AIFM1 and VDAC1 proteins. **(B–E)** Statistical results of the relative expression levels of COX5B, NDUFV2, NUDT5, AIFM1. Data are presented as the mean ± SEM. *p < 0.05, **p < 0.01.

To validate our previous findings from MR and GEO analyses, we next assessed the expression of the four candidate mitochondrial proteins in the hippocampus of VaD rats. Importantly, VDAC1 expression remained stable across the Sham, Model, and Donepezil groups. Coupled with uniform total protein loading confirmed by Ponceau S staining (Supplementary Figures 1A–C), these results validate the reliability of our quantitative Western blot data in this 2-VO model. Following normalization to VDAC1, we observed that the model group had a substantial increase in NUDT5 expression, and a significant decrease in AIFM1, NDUFV2 and COX5B expression compared to the sham group (*p* < 0.05 for all; Figures 5A–E). Notably, while donepezil treatment successfully ameliorated cognitive and pathological deficits, it did not significantly alter the expression of these four proteins compared to the model group.

## 4 Discussion

In this study, we aimed to connect genetic predictions with animal models to identify mitochondrial targets in VaD. An MR screen identified four candidate proteins, with AIFM1 showing the strongest association (*p* = 0.004), but none passed the FDR correction. Consequently, we did not consider these MR signals as definitive causal evidence but as a starting point. Due to the statistical fragility of genetic estimates, particularly in small cohorts, we tested these targets in a 2-VO rat model to verify if they aligned with actual tissue pathology. The *in vivo* validation supported the MR results to varying extents, showing distinct levels of evidence rather than universal validation. By cross-referencing genetic predictions, GEO transcriptomics, and animal tissue protein levels, COX5B was the only target consistently validated across all analyses, consistently acting as a protective factor. NUDT5 exhibited directional alignment between the genetic MR predictions and actual *in vivo* protein expression. While NUDT5 behaved consistently as an risk factor, its corresponding transcriptomic shifts in the GEO cohort did not fully clear strict statistical thresholds, displaying a consistent upward trend but lacked a significant *p*-value. Given the underpowered nature of this specific microarray dataset, we classify NUDT5 as highly promising, *in vivo*-validated targets with partial transcriptomic support. We acknowledge unresolved contradictions with AIFM1 and NDUFV2. AIFM1, a protective signal in MR analysis, was identified as an up-regulated risk gene in GEO and down-regulated *in vivo*. NDUFV2 was an up-regulated risk in MR and GEO but down-regulated in the VaD animal model.

These discrepancies highlight the complexity of biological systems and the need for rigorous validation of genetically prioritized targets. We focus on the most reliable targets and emphasize the importance of COX5B, and NUDT5 dysregulation in understanding VaD pathogenesis. The significant downregulation of COX5B indicates a breakdown of the respiratory chain, as COX5B is essential for the structural integrity of Complex IV, and its loss severely impairs the complex’s function (23–27). Losing both the specific structural and assembly components means the electron transport chain is essentially disabled, inevitably leading to a massive energy crisis and unchecked ROS production. Alongside this structural breakdown, the robust upregulation of NUDT5 reflects a severe metabolic stress response. NUDT5 is a NUDIX hydrolase intimately involved in purine nucleotide metabolism. Under oxidative stress, human NUDT5 hydrolyzes 8-oxo-dGDP sanitize the oxidized nucleotide pool and prevent mutagenic misincorporation into DNA (28–30). Furthermore, recent studies highlight its role in metabolic shifting: when mitochondrial respiration is impaired, NUDT5 actively suppresses energy-demanding *de novo* purine biosynthesis in favor of purine salvage (31). In the ischemic VaD microenvironmen, the significant upregulation of NUDT5 likely initially serves as a compensatory response to conserve energy and clear oxidized nucleotides. However, chronic overexpression can perturb purine-pyrimidine balance and induce replication stress, ultimately exacerbating neurovascular damage.

This study has several methodological limitations. The statistical power of our MR analysis is limited by the small number of VaD cases in the FinnGen database and its mainly European demographic, which affects generalizability. Additionally, using plasma pQTLs to infer central nervous system pathology is problematic, especially for inner membrane respiratory complexes like COX5B. Therefore, we view these MR signals as indicators of systemic metabolic vulnerabilities rather than definitive causal brain mechanisms. To address the plasma-to-brain gap, we used an *in vivo* 2-VO rat model to validate tissue alignment between MR signals (COX5B, NUDT5) and hippocampal expression. Future studies focusing on brain-specific QTLs are needed. Second, regarding the transcriptomic cross-referencing, the GSE122063 dataset is inherently underpowered. Its limited sample size, compounded by the bulk-tissue nature of the microarray that inevitably masks cell-type-specific dynamics, significantly inflates the false-negative rate. This limitation likely obscured actual biological shifts, such as the NUDT5 expression, restricting this cohort’s utility strictly to hypothesis-generating support. Using the MWM test for both baseline validation and post-treatment evaluation poses risks like selection bias, regression to the mean, and practice effects. We minimized practice effects with standardized timelines and a 28-day memory decay interval, but future studies should use different cognitive tasks to avoid these issues entirely. Finally, the observation that donepezil significantly ameliorated VaD symptoms without modifying the identified mitochondrial proteins requires strict interpretation. Rather than acting as direct upstream causal drivers, these specific structural alterations may simply represent parallel epiphenomena, or they are fundamentally not required for the symptomatic recovery pathways mediated by donepezil. Future research must prioritize evaluating fully triangulated targets, specifically COX5B, to determine whether its direct modulation can independently rescue ischemic pathology.

Overall, this study identifies several dysregulated mitochondrial proteins associated with VaD, highlighting their primary value as potential disease-state biomarkers rather than direct therapeutic targets. Future research should clarify how these proteins respond to ischemic injury at the molecular level, and evaluate their levels in patient cerebrospinal fluid or peripheral blood to establish their clinical diagnostic utility. Given that proteins, such as COX5B, are structural subunits of multi-protein respiratory chain complexes, thoroughly mapping their dynamic expression across different disease stages remains a necessary prerequisite before exploring any pharmacological interventions.

## Data Availability

The datasets presented in this study can be found in online repositories. The names of the repository/repositories and accession number(s) can be found in the article/Supplementary material.
