# Supplementary material for: Mitochondrial protein alterations in vascular dementia: evidence from Mendelian randomization, transcriptomics, and a chronic hypoperfusion model
Source: Front Neurol. 2026 Jul 9;17:1794851. doi: 10.3389/fneur.2026.1794851 (PMC13391407; doi:10.3389/fneur.2026.1794851)
Supplement: Supplementary file 1 [file Table_1.DOCX]

**Supplementary Table 1**. p-values and FDR-adjusted p-values for the 66 mitochondrial proteins identified in the MR analysis.

| Proteins | P value | FDR (Benjamini-Hochberg) | Cochran's Q (IVW) | Cochran's Q *p*-value (IVW) | MR-Egger Intercept | Egger Intercept *p*-value |
| --- | --- | --- | --- | --- | --- | --- |
| prot-a-1055 | 0.480 | 0.873 | 5.840 | 0.756 | -0.012 | 0.631 |
| prot-a-1220 | 0.296 | 0.857 | 15.857 | 0.257 | -0.011 | 0.655 |
| prot-a-1281 | 0.062 | 0.677 | 4.714 | 0.581 | -0.020 | 0.581 |
| prot-a-1339 | 0.282 | 0.857 | 15.266 | 0.227 | 0.048 | 0.046 |
| prot-a-1356 | 0.238 | 0.857 | 10.487 | 0.399 | -0.019 | 0.538 |
| prot-a-1368 | 0.430 | 0.873 | 4.235 | 0.237 | 0.041 | 0.460 |
| prot-a-1392 | 0.216 | 0.857 | 5.841 | 0.558 | 0.030 | 0.663 |
| prot-a-1572 | 0.150 | 0.857 | 8.482 | 0.582 | 0.010 | 0.716 |
| prot-a-1761 | 0.254 | 0.857 | 10.697 | 0.469 | -0.003 | 0.912 |
| prot-a-1783 | 0.522 | 0.873 | 8.688 | 0.796 | 0.010 | 0.726 |
| prot-a-1864 | 0.289 | 0.857 | 23.733 | 0.022 | 0.031 | 0.449 |
| prot-a-1907 | 0.175 | 0.857 | 5.584 | 0.849 | 0.009 | 0.728 |
| prot-a-1940 | 0.916 | 0.959 | 5.037 | 0.411 | 0.064 | 0.192 |
| prot-a-1941 | 0.618 | 0.873 | 3.098 | 0.928 | -0.012 | 0.755 |
| prot-a-1942 | 0.277 | 0.857 | 10.550 | 0.394 | -0.017 | 0.374 |
| prot-a-1943 | 0.517 | 0.873 | 11.216 | 0.129 | -0.052 | 0.174 |
| prot-a-1944 | 0.508 | 0.873 | 19.216 | 0.023 | -0.107 | 0.126 |
| prot-a-1945 | 0.624 | 0.873 | 6.484 | 0.839 | 0.005 | 0.818 |
| prot-a-1953 | 0.447 | 0.873 | 14.589 | 0.148 | 0.034 | 0.455 |
| prot-a-1961 | 0.692 | 0.873 | 7.291 | 0.399 | 0.016 | 0.653 |
| prot-a-1964 | 0.714 | 0.873 | 12.002 | 0.285 | 0.012 | 0.755 |
| prot-a-1965 | 0.396 | 0.873 | 7.207 | 0.616 | 0.026 | 0.370 |
| prot-a-1969 | 0.706 | 0.873 | 10.652 | 0.473 | -0.028 | 0.316 |
| prot-a-1970 | 0.981 | 0.981 | 8.138 | 0.774 | -0.012 | 0.587 |
| prot-a-1997 | 0.932 | 0.959 | 4.567 | 0.713 | -0.032 | 0.592 |
| prot-a-2022 | 0.204 | 0.857 | 12.061 | 0.601 | -0.010 | 0.668 |
| prot-a-2024 | 0.702 | 0.873 | 14.730 | 0.195 | -0.019 | 0.571 |
| prot-a-2025 | 0.830 | 0.928 | 27.301 | 0.054 | -0.001 | 0.961 |
| prot-a-2026 | 0.048 | 0.628 | 3.423 | 0.635 | -0.022 | 0.483 |
| prot-a-203 | 0.944 | 0.959 | 12.744 | 0.310 | -0.001 | 0.975 |
| prot-a-2041 | 0.691 | 0.873 | 8.573 | 0.661 | -0.030 | 0.314 |
| prot-a-2128 | 0.313 | 0.857 | 16.861 | 0.077 | 0.036 | 0.287 |
| prot-a-2129 | 0.021 | 0.628 | 11.348 | 0.658 | 0.022 | 0.256 |
| prot-a-2190 | 0.312 | 0.857 | 21.207 | 0.020 | -0.029 | 0.566 |
| prot-a-2235 | 0.687 | 0.873 | 6.205 | 0.624 | -0.023 | 0.321 |
| prot-a-2236 | 0.671 | 0.873 | 1.575 | 0.665 | 0.034 | 0.570 |
| prot-a-2454 | 0.817 | 0.928 | 10.502 | 0.232 | 0.021 | 0.681 |
| prot-a-2526 | 0.564 | 0.873 | 5.591 | 0.848 | -0.024 | 0.314 |
| prot-a-2575 | 0.674 | 0.873 | 25.431 | 0.044 | -0.018 | 0.476 |
| prot-a-2627 | 0.238 | 0.857 | 12.810 | 0.235 | 0.029 | 0.548 |
| prot-a-2653 | 0.544 | 0.873 | 7.659 | 0.662 | -0.027 | 0.384 |
| prot-a-2657 | 0.774 | 0.922 | 4.646 | 0.703 | -0.034 | 0.361 |
| prot-a-2737 | 0.583 | 0.873 | 10.360 | 0.664 | 0.078 | 0.061 |
| prot-a-2749 | 0.810 | 0.928 | 14.096 | 0.169 | -0.015 | 0.610 |
| prot-a-2764 | 0.465 | 0.873 | 14.990 | 0.091 | 0.014 | 0.714 |
| prot-a-2776 | 0.271 | 0.857 | 14.694 | 0.327 | 0.023 | 0.357 |
| prot-a-2799 | 0.932 | 0.959 | 19.806 | 0.048 | -0.013 | 0.718 |
| prot-a-2866 | 0.189 | 0.857 | 34.795 | 0.001 | -0.010 | 0.835 |
| prot-a-300 | 0.438 | 0.873 | 18.780 | 0.536 | 0.003 | 0.853 |
| prot-a-3015 | 0.342 | 0.868 | 3.127 | 0.926 | 0.011 | 0.724 |
| prot-a-308 | 0.943 | 0.959 | 9.434 | 0.665 | -0.060 | 0.049 |
| prot-a-332 | 0.618 | 0.873 | 10.836 | 0.211 | 0.061 | 0.039 |
| prot-a-385 | 0.199 | 0.857 | 11.596 | 0.170 | 0.068 | 0.025 |
| prot-a-534 | 0.161 | 0.857 | 17.038 | 0.030 | -0.050 | 0.240 |
| prot-a-612 | 0.041 | 0.628 | 17.058 | 0.315 | -0.083 | 0.012 |
| prot-a-63 | 0.004 | 0.285 | 15.103 | 0.236 | -0.015 | 0.486 |
| prot-a-637 | 0.437 | 0.873 | 11.186 | 0.191 | 0.043 | 0.059 |
| prot-a-638 | 0.043 | 0.628 | 11.650 | 0.391 | 0.020 | 0.399 |
| prot-a-64 | 0.325 | 0.857 | 1.166 | 0.884 | -0.015 | 0.939 |
| prot-a-640 | 0.563 | 0.873 | 10.891 | 0.695 | 0.013 | 0.529 |
| prot-a-641 | 0.848 | 0.933 | 7.421 | 0.593 | 0.007 | 0.763 |
| prot-a-818 | 0.712 | 0.873 | 21.482 | 0.205 | -0.046 | 0.072 |
| prot-a-825 | 0.305 | 0.857 | 25.487 | 0.183 | 0.003 | 0.883 |
| prot-a-847 | 0.782 | 0.922 | 13.812 | 0.055 | 0.033 | 0.655 |
| prot-a-896 | 0.475 | 0.873 | 22.416 | 0.097 | -0.039 | 0.279 |
| prot-a-992 | 0.491 | 0.873 | 10.670 | 0.638 | 0.027 | 0.313 |

**Supplementary Table 2.** Multi-level evidence summary for the four candidate mitochondrial proteins in VaD.

|  | GEO analysis  (VaD *vs* Control) | | | MR analysis | | | | | | Western Blot | Summary |
| --- | --- | --- | --- | --- | --- | --- | --- | --- | --- | --- | --- |
|  | Log_2_FC | FDR | Expression Patterns in VaD | B | *p*val | OR | OR_LCI95 | OR_UCI95 | MR Causal Estimate | Expression Patterns in VaD | Multi-omics Consistency |
| NDUFV2 | 1.134 | 0.049 | Significantly Up-regulated | 0.143881429449544 | 0.0475833745603456 | 1.15474718139838 | 1.00153147814523 | 1.33140203982099 | Increased Risk | Significantly Down-regulated | Inconsistent |
| COX5B | -1.351 | 0.038 | Significantly Down-regulated | -0.118709483 | 0.042909669910765 | 0.888065761770336 | 0.791653162220625 | 0.996220106058183 | Reduced Risk | Significantly Down-regulated | Consistent |
| NUDT5 | 1.104 | 0.071 | Up-regulation trend | 0.114500702314618 | 0.0209855586456431 | 1.1213134285785 | 1.01742476099349 | 1.23581010932219 | Increased Risk | Significantly Up-regulated | Consistent |
| AIFM1 | 1.154 | 0.038 | Significantly Up-regulated | -0.133169509 | 0.00431470231965505 | 0.875316705733674 | 0.798821009879165 | 0.959137686491683 | Reduced Risk | Significantly Down-regulated | Inconsistent |


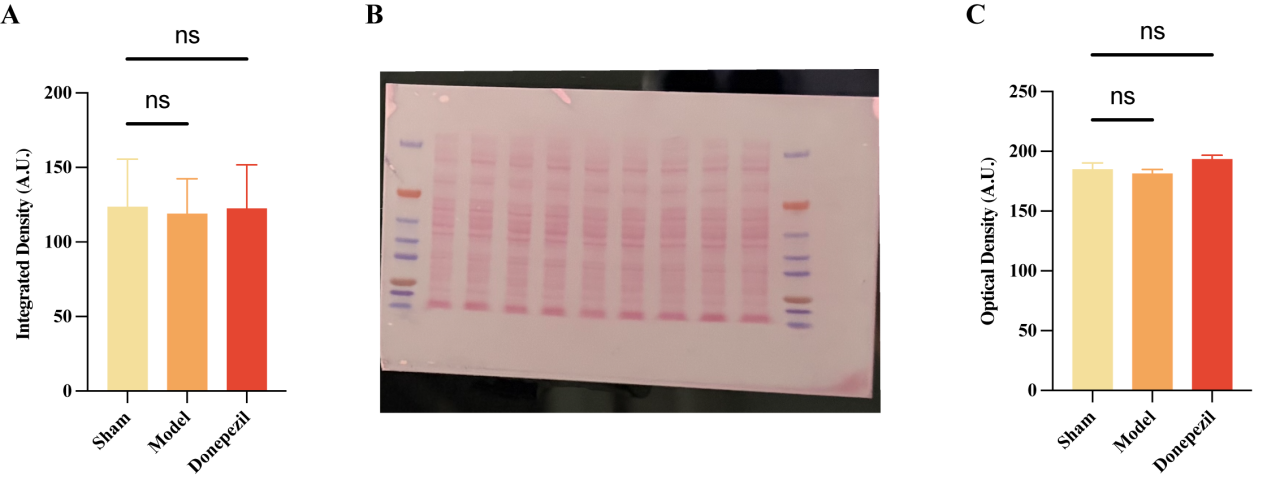


**Supplementary Figure 1**. Validation of protein loading consistency across experimental groups. (A) Quantitative analysis of VDAC1 protein levels among the three groups. (B) Representative blot images of total protein staining using Ponceau S. (C) Quantification of total protein loading, determined by measuring the integrated density of the Ponceau S staining. The Y-axis represents the total protein intensity in arbitrary units (A.U.). Data are presented as mean ± SD. ns indicates no significant difference (*p* > 0.05).
